# Colorectal Cancer Burden and Trends in South-West Oltenia, Romania: A 15-Year Real-World Study from a Regional Referral Cancer Center

**DOI:** 10.3390/cancers18162615

**Published:** 2026-08-13

**Authors:** Tradian Ciprian Berisha, Florin Burada, Mihai Gabriel Cucu, Ana-Maria Ciurea, Alina Maria Mehedinteanu, Puiu Olivian Stovicek, Ramona Adriana Schenker, Michael Schenker, Monica-Laura Cara

**Affiliations:** 1 Doctoral School, University of Medicine and Pharmacy Craiova, 200349 Craiova, Romania; ciprian.berisha@sfnectarie.ro; 2 Sf. Nectarie Oncology Center, 200347 Craiova, Romania; ana.ciurea@umfcv.ro (A.-M.C.); alina.mehedinteanu@sfnectarie.ro (A.M.M.); olivian.stovicek@sfnectarie.ro (P.O.S.); ramona.schenker@sfnectarie.ro (R.A.S.); monica.cara@umfcv.ro (M.-L.C.); 3 Human Genomics Laboratory, University of Medicine and Pharmacy of Craiova, 200349 Craiova, Romania; florin.burada@umfcv.ro; 4 Regional Center of Medical Genetics Dolj, Emergency Clinical County Hospital Craiova, 200642 Craiova, Romania; 5 Department of Oncology, Faculty of Medicine, University of Medicine and Pharmacy of Craiova, 200349 Craiova, Romania; 6 Department of Pharmacology, Faculty of Nursing, Târgu Jiu Subsidiary, “Titu Maiorescu” University, 040441 Bucharest, Romania; 7 Department of Public Health, Faculty of Medicine, University of Medicine and Pharmacy of Craiova, 200349 Craiova, Romania

**Keywords:** colorectal cancer, socio-demographic, epidemiology, cancer staging, health disparities, real-world data

## Abstract

**Background/Objectives:** Colorectal cancer (CRC) remains a major contributor to the global oncological burden, with marked disparities in mortality and survival between Western and Eastern European countries. Romania continues to report unfavorable CRC outcomes, driven by delayed diagnosis, limited screening uptake, and heterogeneous care pathways. This study aimed to assess the 15-year institutional description of colorectal cancer case-mix in South-West Oltenia, Romania, using real-world data from a high-volume oncology referral center. **Methods:** We conducted a retrospective observational study including 3497 patients with newly diagnosed CRC registered between 2011 and 2025. Sociodemographic characteristics, tumor localization, stage at diagnosis, and histopathological grade were analyzed. Temporal patterns were assessed across three five-year intervals. **Results:** The institutional CRC case volume increased, with 434 cases recorded in 2011–2015, 1015 in 2016–2020, and 2048 in 2021–2025. Male patients accounted for 60.5% and urban residents for 60.3% of cases. Early-onset CRC was identified in 12.0% of patients, with no significant temporal trend. Rectosigmoid junction/rectal cancers represented the largest anatomical group, followed by left-sided cancers, with stable distribution across periods. Advanced stage disease was frequent, with 77.1% of patients diagnosed in stage III–IV, increasing from 67.7% to 82.0% across study intervals (*p* < 0.001). Histopathological grade changed significantly (*p* < 0.001), due to a progressive increase in unspecified grade, from 16.1% to 45.5%. **Conclusions:** Colorectal cancer represents a substantial institutional workload at this regional referral center, emphasizing the need for strengthened early detection strategies, optimized referral system, improved diagnostic access, and standardized pathological reporting practices.

## Simple Summary

Colorectal cancer is one of the most common cancers worldwide, and patients in Eastern Europe survive it less often than those in Western Europe. Late diagnosis is thought to be part of the reason, but very little has been published about how patients in south-western Romania present with this disease. We reviewed the records of 3497 patients treated at a regional cancer center over fifteen years to describe who they were, where their tumors were, and how far the disease had spread when they were referred. Most were men, most lived in towns, and more than three quarters arrived with disease that had already spread. Pathology reports increasingly did not state how aggressive the tumor looked under the microscope. These findings describe patients referred to one center rather than the whole region, but they give other researchers a starting point for a part of Europe with almost no published data.

## 1. Introduction

Gastrointestinal malignancies, including colorectal, gastric, pancreatic, hepatic, and esophageal cancers, constitute a major component of the global cancer burden. According to the Global Cancer Statistics 2022 report, there were close to 20 million new cancer cases and 9.7 million cancer deaths worldwide, with gastrointestinal cancers accounting for approximately 24.6% of all new cases and 33.2% of all cancer-related deaths [1,2].

Colorectal cancer is the third most diagnosed cancer worldwide and ranks second in cancer mortality, while gastric, liver, pancreatic, and esophageal cancers together contribute disproportionately to cancer deaths because of their aggressive biological behavior and the predominance of late-stage presentation at diagnosis [1,2]. Incidence rates continue to rise driven by population growth, aging, and sustained exposure to modifiable risk factors, with gastrointestinal cancer burden projected to nearly double by 2050 [2]. These cancers also show marked geographic and socioeconomic disparities, with Eastern European countries generally reporting higher mortality and poorer survival outcomes than their Western European counterparts, a pattern linked to differences in screening implementation, diagnostic infrastructure, and access to specialized care [1,3].

The development of gastrointestinal cancers is shaped by a complex interplay of environmental exposures, lifestyle behaviors, infectious agents, and host-related susceptibility, with a substantial proportion of cases attributable to modifiable factors [1,4,5]. Tobacco, smoking and alcohol consumption are strongly associated with cancers of the esophagus, stomach, liver, pancreas, and colorectum, often acting through synergistic carcinogenic mechanisms [1]. High consumption of red and processed meat together with low dietary fibre intake increases colorectal cancer risk, as demonstrated in large-scale meta-analyses of prospective cohort studies [6]. Obesity further contributes to carcinogenesis through metabolic dysregulation, chronic low-grade inflammation, hyperinsulinemia, and activation of the insulin-like growth factor axis, mechanisms that have been consistently linked to colorectal and other gastrointestinal cancers [7]. Infectious agents play a central etiological role in several gastrointestinal malignancies: *Helicobacter pylori* are classified as a Group I carcinogen and is the primary cause of gastric adenocarcinoma, collectively responsible for the largest share of infection-attributable cancers worldwide [4,8,9]. Chronic infection with hepatitis B and C viruses substantially increases the risk of hepatocellular carcinoma through progressive liver injury, fibrosis, and cirrhosis, together accounting for more than 70% of liver cancer cases globally. Human papillomavirus is similarly implicated as the dominant etiological factor in anal and selected lower gastrointestinal cancers [4]. Host-related factors further modulate susceptibility: hereditary syndromes including Lynch syndrome and familial adenomatous polyposis account for 2–5% of all colorectal cancers and confer markedly elevated lifetime risk, with familial adenomatous polyposis approaching a near-100% risk of colorectal cancer without prophylactic colectomy [10]. Chronic inflammatory conditions, including inflammatory bowel disease, cirrhosis, Barrett’s esophagus, and chronic pancreatitis, may promote carcinogenesis through sustained tissue injury, oxidative stress, and dysregulated cellular regeneration [11].

Despite advances in diagnostic and therapeutic approaches, the burden of gastrointestinal cancers remains unevenly distributed across Europe. Western European countries have achieved notable improvements in cancer survival, largely attributable to the implementation of organized screening programs and increased public awareness. In contrast, Eastern European countries continue to face higher mortality rates and less favourable outcomes, reflecting delayed diagnosis and limited access to specialized care [3]. The EUROCARE-5 study, which analyzed data from more than 10 million patients across 29 European countries, documented persistent and substantial survival differences between Eastern and Western Europe for colorectal, gastric, and other gastrointestinal cancers; these differences were not explained by demographic confounders alone [3].

Romania illustrates this disparity. Although colorectal cancer incidence is not consistently higher than the European average, mortality remains disproportionately elevated, pointing to persistent weaknesses in early detection, timely diagnosis, and access to effective treatment. Romania has among the highest avoidable mortality from colorectal cancer in the European Union(EU), with rates 57% above the EU average among men and 32% above the EU average among women, underscoring the magnitude of this structural gap [12].

The absence of long-standing, fully operational screening programs has likely contributed to a diagnostic pattern dominated by symptomatic presentation at advanced stages. Until 2024, Romania was one of only two EU Member States without a national colorectal cancer screening program [13,14]. The first organized pilot program implemented through the ROCCAS project in four regions between 2022 and 2023, showed that while fecal immunochemical test (FIT) return rates could reach 90%; however, colonoscopy uptake among FIT-positive individuals was only 51.3%, revealing a critical bottleneck in the screening cascade [15]. At the same time, demographic changes and sustained exposure to lifestyle risk factors, including shifts in dietary patterns, rising obesity rates, and high rates of alcohol and tobacco consumption, may contribute to a sustained colorectal cancer burden and may influence patterns of presentation at regional oncology referral centers [1].

In recent years, attention has been paid to changes in the epidemiology of colorectal cancer, including a rising incidence among younger adults and shifts in tumor localization. This trend has been reported in several European countries, with incidence rates in adults under 50 years increasing steadily over the past two decades [16,17]. However, these trends are not universal and may differ substantially according to regional healthcare structures, population characteristics, and the maturity of screening program implementation [5].

In this context, detailed regional data is essential. Although national statistics provide valuable information, they may obscure local variations that are critical for understanding disease dynamics and guiding targeted interventions.

South-West Oltenia is a Nomenclature of Territorial Units for Statistics, level 2 (NUTS-2) development region located in south-western Romania, comprising five counties—Dolj, Gorj, Mehedinți, Olt, and Vâlcea—with an area of approximately 29,212 km^2^ and an estimated population of about 1.86 million inhabitants in 2024. Characterized by a mixed urban and rural population, the region faces potential disparities in healthcare access and diagnostic pathways that may influence cancer outcomes. The “Sf. Nectarie” Oncology Center in Craiova is the largest oncology referral facility in the region, providing day-hospital oncology care, systemic treatment, supportive care, psychological counselling, and radiotherapy services.

Against this background, the present study aimed to provide a 15-year real-world assessment of institutional colorectal cancer case volume and clinicopathological patterns in South-West Oltenia, Romania, by analysing demographic, anatomic and clinicopathological patterns among patients treated in the largest regional oncology referral center, with particular attention to temporal trends in late-stage disease presentation and to findings relevant for early detection, referral pathways, and regional cancer care planning.

## 2. Materials and Methods

### 2.1. Study Design and Setting

This was a retrospective, observational, real-world study conducted at the “Sf. Nectarie” Oncology Center in Craiova, Romania, a high-volume regional oncology referral facility serving the South-West Oltenia region. South-West Oltenia was one of the four Romanian regions included in the ROCCAS pilot program, and the regional screening center was hosted by the University of Medicine and Pharmacy of Craiova [15].

The center provides oncological care for a heterogeneous population from both urban and rural areas, offering a relevant institutional perspective on regional colorectal cancer patterns. The study included patients newly diagnosed with and treated for colorectal cancer over a 15-year period, from January 2011 to December 2025.

The analysis was designed to evaluate temporal changes in institutional colorectal cancer burden, demographic characteristics, tumor localization, stage at diagnosis, and histopathological grading using institutional data from a regional oncology referral center.

### 2.2. Regional Colorectal Cancer Screening Context

Organized colorectal cancer screening became operational in South-West Oltenia only during the final years of the study period. The ROCCAS program targeted adults aged 50–74 years using risk assessment and FIT, followed by colonoscopy after a positive result. Screening records were maintained separately from the national cancer registry and from the institutional oncology database; therefore, screen-detected cases could not be identified in the present cohort [15].

### 2.3. Cancer Registration and Case Ascertainment

Cancer registration in Romania has been regulated through regional population-based registries since Ministry of Health Order no. 2027/2007, while the National Cancer Registry was established by Government Decision no. 663/2023 [18,19]. Because no continuously operating population-based registry covered South-West Oltenia throughout 2011–2025, this study used the center’s hospital-based oncology record. All eligible patients treated at the center were registered irrespective of county of residence; consequently, the results represent institutional distributions rather than population rates.

### 2.4. Data Quality

Data quality was considered in terms of comparability, validity, completeness, and timeliness [20,21]. All included cases had histopathological confirmation, and duplicate records were removed by cross-checking patient identifiers, diagnosis date, and tumor site.

Histopathological examinations were performed in external laboratories, in the surgical and endoscopy units where the diagnostic material was obtained; the study center does not operate its own pathology service. Reports were presented at the center in printed form, complete reports were a requirement for registration, and were requested again from the referring unit where the document initially presented was incomplete. Standardized synoptic reporting templates for colorectal carcinoma were not in routine use across the referring laboratories during the study period, and reports were issued in free-text form according to the conventions of each unit. Cases recorded as unspecified grade therefore correspond to reports in which the issuing laboratory did not state a differentiation grade, rather than to incomplete transfer of the pathology document to the center.

Tumors coded as colon, unspecified (11.0%) and cases with unspecified grade (39.0%) were retained as reported and were not imputed. Population-level completeness could not be evaluated because a regional population-based denominator and an independent complete registry were unavailable.

### 2.5. Study Population

All consecutive patients with newly diagnosed colorectal cancer registered at the center during the study period were considered eligible for inclusion. Colorectal cancer was defined as a malignant tumor originating in the colon, rectosigmoid junction and rectum, according to the 10th edition of the International Statistical Classification of Diseases and Related Health Problems (ICD-10). For the identification of colorectal cancer cases, the following ICD-10 codes were considered: C18, malignant neoplasm of the colon; C19, malignant neoplasm of the rectosigmoid junction; and C20, malignant neoplasm of the rectum.

Only cases with histopathological confirmation and sufficient diagnostic documentation were included in the final analysis. Diagnosis was based on available clinical, endoscopic, imaging, surgical, and histopathological records from the institutional database.

Patients with recurrent disease initially diagnosed before the study period, metastatic lesions originating from non-colorectal primary tumors, duplicate records, or incomplete diagnostic documentation were excluded.

### 2.6. Data Collection and Variables

Data were collected retrospectively from institutional electronic medical records, oncology registration files, pathology reports, and available clinical documentation. The following variables were extracted: year of diagnosis, sex, age at diagnosis, area of residence, tumor localization, stage at diagnosis, and histopathological grade. Extracted data were cross-checked against available clinical and pathology documentation when necessary.

Area of residence was classified as urban or rural according to the patient’s recorded home address.

Early-onset colorectal cancer was defined as colorectal cancer diagnosed before the age of 50 years. Patients were categorized into two age groups: early-onset cases, defined as patients aged < 50 years at diagnosis, and later-onset cases, defined as patients aged ≥ 50 years at diagnosis.

Tumor localization was classified according to anatomical subsite. Right-sided colon cancers included tumors of the cecum, ascending colon, hepatic flexure, and transverse colon. Left-sided colon cancers included tumors of the splenic flexure, descending colon, sigmoid colon, and rectosigmoid junction. Rectal cancer was analyzed separately when documented as a distinct anatomical subsite.

Disease stage at diagnosis was classified according to American Joint Committee on Cancer (AJCC) TNM Staging System, based on the staging information available in the medical records. Cases were grouped into stages I, II, III, and IV. For additional analysis, stage was dichotomized into early-stage disease, defined as stage I–II, and advanced-stage disease, defined as stage III–IV.

The anatomic subsites of the colon (left, right) and rectum were categorized according to the International Classification of Diseases for Oncology, 3rd edition ICD-O-3, topography codes. Right-sided colon cancers were identified using the following topography codes: cecum (code C18.0), ascending colon (code C18.2), hepatic flexure (code C18.3), and transverse colon (code C18.4). Left-sided colon cancers were identified using the topography codes: splenic flexure (code C18.5), descending colon (code C18.6), sigmoid colon (code C18.7). Rectosigmoid junction/rectal cancer included tumors coded as rectosigmoid junction (C19.9) and rectum (C20.9).

Histopathological grade was recorded as stated in the original pathology report, using the categories well differentiated, moderately differentiated, poorly differentiated, undifferentiated, or unspecified. The original slides were not reviewed, and tumors were not retrospectively regraded. Because the reports originated from different pathology services over the 15-year study period, uniform application of a single WHO or CAP grading system could not be confirmed. For the present analysis, we grouped well- and moderately differentiated tumors as low grade and poorly differentiated and undifferentiated tumors as high grade. Cases without a reported grade were classified as unspecified and were not imputed or reassigned.

### 2.7. Temporal Grouping

For temporal analysis, the 15-year study period was divided into three predefined consecutive five-year intervals: 2011–2015, 2016–2020, and 2021–2025. The grouping was defined a priori, before analysis, and was not selected on the basis of the results obtained. It was used to compare demographic and clinicopathological characteristics over time and to evaluate temporal patterns in disease presentation, providing stable estimates in the earliest years of the period, when annual case numbers at the center were small and its regional referral role was still developing.

Temporal trends were assessed using the annual absolute number of newly registered colorectal cancer cases and, where applicable, the yearly proportion of colorectal cancer cases among all malignancies recorded annually at the regional referral cancer center. These indicators were interpreted as measures of institutional case volume and clinical presentation patterns rather than population-based incidence.

### 2.8. Outcomes

The primary outcomes of the study were the temporal evolution of colorectal cancer case volume and the distribution of demographic and clinicopathological characteristics over the 15-year period. Attention was given to advanced-stage disease, defined as stage III–IV, as one of the key clinicopathological indicators.

Secondary outcomes included temporal changes in sex distribution, age at diagnosis, area of residence, tumor localization, early-onset colorectal cancer, stage group, and histopathological grading. Early-onset colorectal cancer was analyzed overall and across the three predefined five-year intervals. The study also assessed the proportion of tumors without clearly defined histopathological grading as an indicator of variability in pathological reporting over time.

### 2.9. Statistical Analysis

Categorical variables, including sex, area of residence, tumor localization, tumor grade, early-onset status, and stage at diagnosis, were summarized as frequencies and percentages. Continuous variables were reported as mean ± standard deviation or median and interquartile range, according to data distribution.

Comparisons between the three predefined study periods were performed using the Pearson chi-square test or Fisher’s exact test for categorical variables, as appropriate. Ordered temporal trends in binary or categorical variables were assessed using the chi-square test for linear trend (linear-by-linear association test, SPSS). This analysis was used to evaluate ordered changes across the three study periods, particularly in the proportion of patients diagnosed with advanced-stage disease, early-onset colorectal cancer, and unspecified histopathological grade. A two-sided *p*-value < 0.05 was considered statistically significant.

All statistical analyses were performed using IBM SPSS Statistics, version 22.0 (IBM Corp., Armonk, NY, USA). The analyses were descriptive and unadjusted. Multivariable modelling was not undertaken because the institutional record did not contain the individual-level variables required to adjust adequately for confounding.

### 2.10. Ethical Considerations

The study was conducted in accordance with the principles of the Declaration of Helsinki and was approved by the Ethics Committee of the University of Medicine and Pharmacy of Craiova, Romania (approval no. 245/25.10.2023, date 25 October 2023) as a retrospective observational investigation.

In accordance with the hospital’s standard admission procedures and applicable national legislation, all patients signed a general informed consent form at each hospitalization, which includes authorization for the use of anonymized clinical data for scientific research purposes. Because the analysis was based exclusively on retrospectively collected, fully anonymized data, the Ethics Committee waived the requirement for additional study-specific informed consent.

All data were handled in compliance with applicable data protection regulations, and no directly identifiable personal information was included at any stage of the analysis.

## 3. Results

### 3.1. Overall Colorectal Cancer Case Volume and Temporal Evolution

During the 15-year study period, 3497 patients with newly diagnosed colorectal cancer admitted for treatment in our referral center were included in the final analysis. When grouped into three consecutive five-year intervals, 434 cases (12.4%) were recorded in 2011–2015, 1015 cases (29.0%) in 2016–2020, and 2048 cases (58.6%) in 2021–2025, indicating an increase in institutional colorectal cancer case volume over time (Table 1).

### 3.2. Demographic Profile: Sex, Age, and Area of Residence and Early- Onset Colorectal Cancer

The baseline demographic and clinicopathological of the study population are presented in Table 2.

The cohort included 2115 male patients (60.5%) and 1382 female patients (39.5%). Patients from urban areas accounted for 2108 cases (60.3%), whereas rural residents represented 1389 cases (39.7%). Early-onset colorectal cancer, defined as diagnosis before 50 years of age, was identified in 421 patients (12.0%) (Table 2).

Across the analyzed period, several consistent patterns emerged among colorectal cancer cases managed in our regional oncology center, outlining the clinicopathological profile of the study population.

#### 3.2.1. Sex Distribution

**Figure 1:**
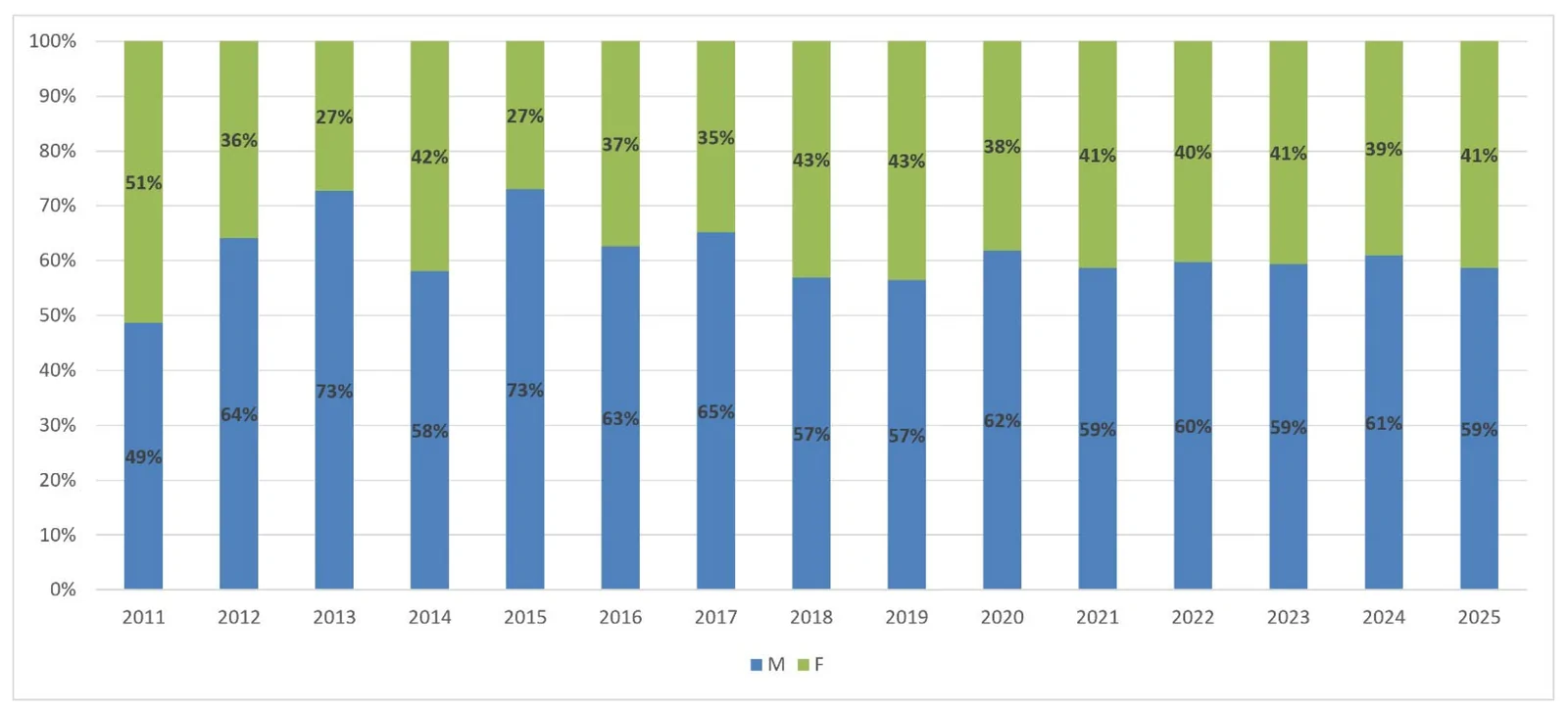
Gender distribution of colorectal cancer cases in institutional cohort, 2011–2025.

Male patients accounted for 2115 cases (60.5%), whereas female patients represented 1382 cases (39.5%). As shown in Figure 1, male patients represented most cases in most years, despite moderate year-to-year fluctuations. After 2012, men generally accounted for approximately 57–73% of annual colorectal cancer cases.

Sex distribution remained relatively stable across the three study periods, with no statistically significant difference between intervals (chi-square test, *p* = 0.134).

#### 3.2.2. Distribution of Cases by Area of Residence

**Figure 2:**
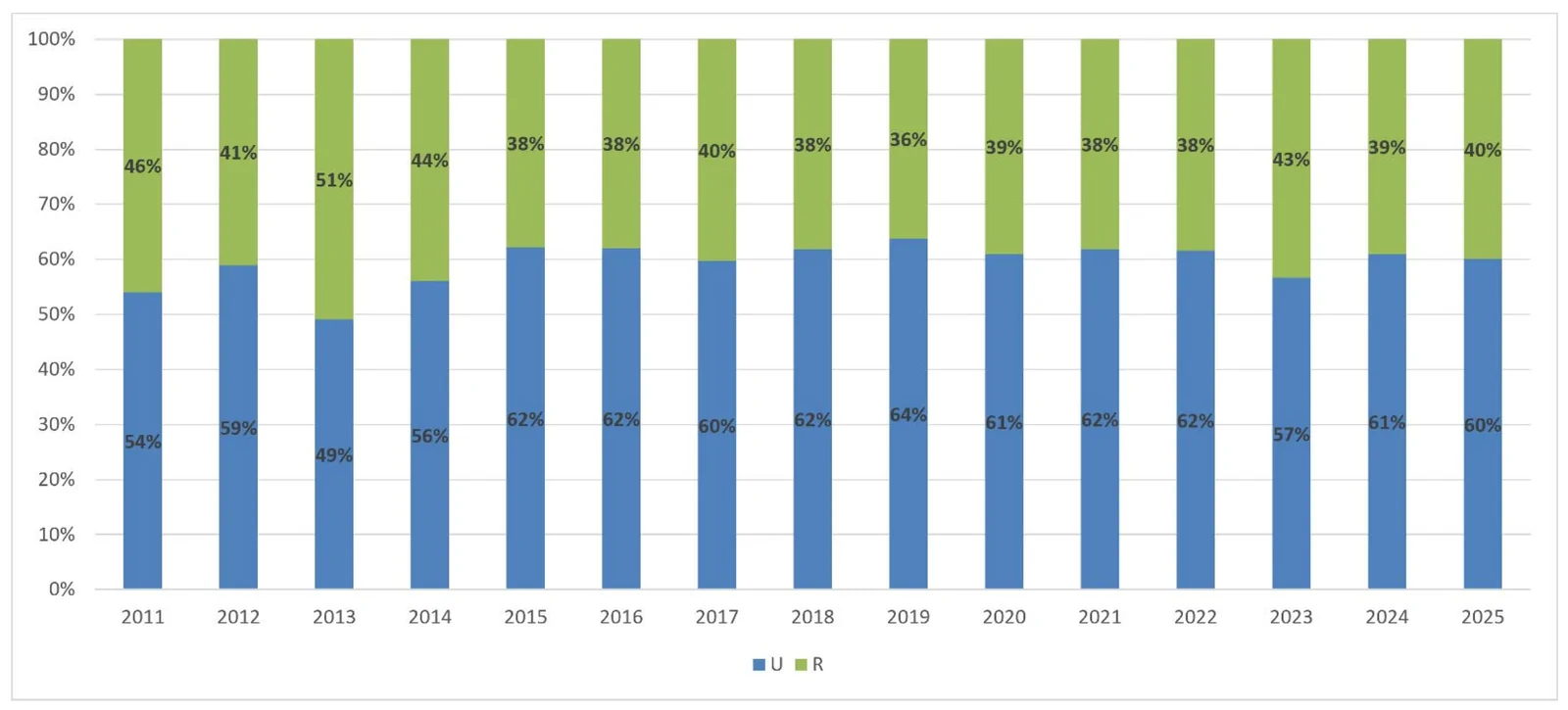
Distribution of colorectal cancer cases by area of residence in institutional cohort, 2011–2025.

Patients from urban areas accounted for most cases, with annual proportions generally close to 60% over the study period (Figure 2). This distribution was similar across the three study periods: urban cases represented 57.4% of patients in 2011–2015, 61.7% in 2016–2020, and 60.2% in 2021–2025.

No statistically significant difference in area of residence was observed across the three intervals (chi-square test, *p* = 0.307). Although minor year-to-year variations were observed, no sustained shift in the urban–rural distribution was identified. This pattern raises the possibility that the observed differences may reflect disparities in access to diagnostic services, screening programs, and healthcare infrastructure for rural population, rather than true differences in disease incidence.

#### 3.2.3. Early-Onset Colorectal Cancer

Early-onset colorectal cancer was identified in 421 patients, representing 12.0% of the overall cohort. Its proportion remained almost similar across the three study periods: 11.1% (n= 48) in 2011–2015, 13.4% (n = 126) in 2016–2020 and 11.6% (n = 237), with no significant difference between intervals (chi-square test, *p* = 0.274) and no significant linear trend over time (*p* for trend = 0.696).

### 3.3. Anatomical Distribution of CCR

**Figure 3:**
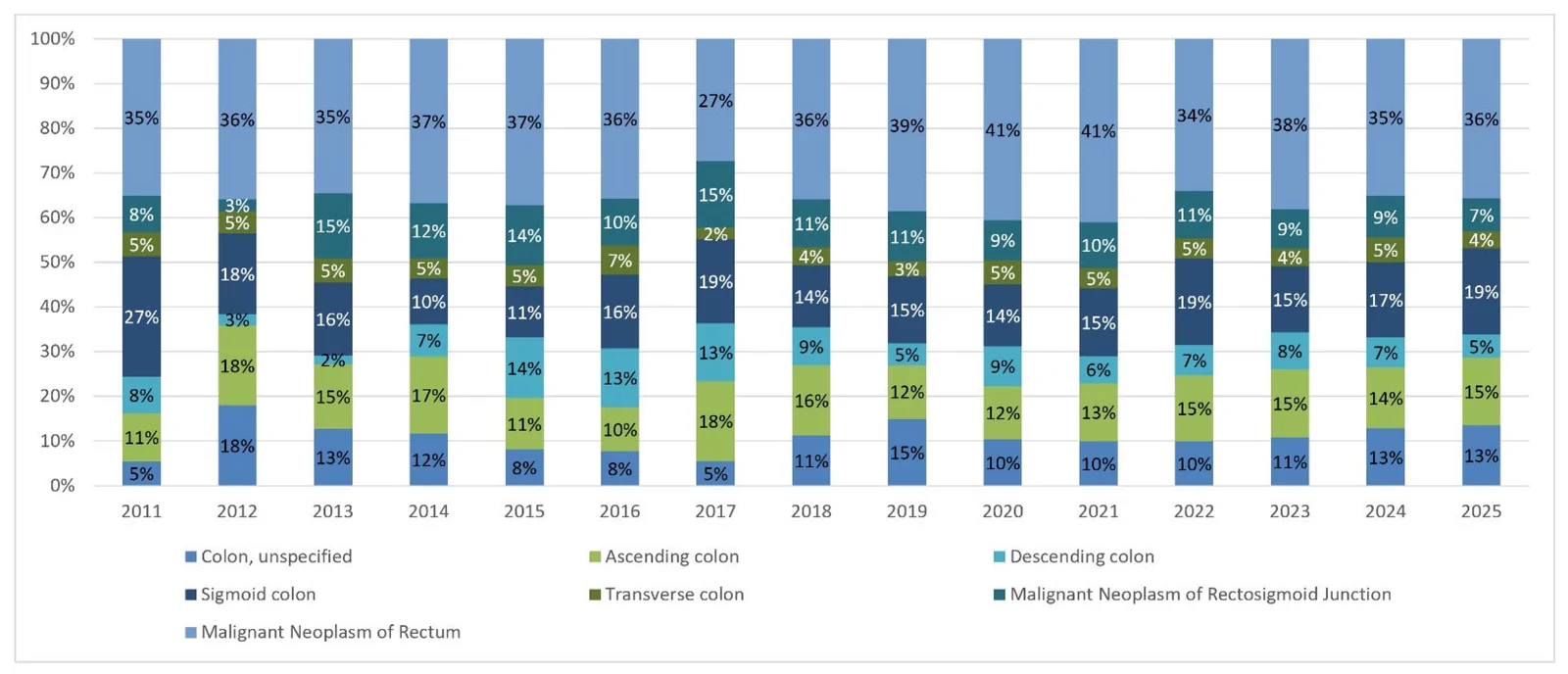
Distribution of colorectal cancer cases by anatomical site in institutional cohort, 2011–2025.

The anatomical distribution of colorectal cancer cases varied between years but showed no significant change across the three study periods (Figure 3). Rectal cancer was the most frequent anatomical site in most years, accounting for 27–41% of annual cases, followed by sigmoid colon cancer, which represented 10–27%. These findings indicate a persistent predominance of distal colorectal tumors. Ascending, transverse, descending, and rectosigmoid tumors accounted for smaller and more variable proportions, while cases classified as “colon, unspecified” fluctuated over time, suggesting possible variability in diagnostic documentation or reporting.

When grouped into right-sided colon, left-sided colon, and rectosigmoid junction/rectal cancer, the latter category accounted for the largest proportion of cases overall, with 1619 patients (46.3%), followed by left-sided colon cancer in 1222 patients (34.9%) and right-sided colon cancer in 656 patients (18.8%). This distribution remained similar across the three study periods, with no statistically significant difference between intervals (chi-square test, *p* = 0.666). (Table 3).

### 3.4. Stage Distribution and Disease Severity at Diagnosis

**Figure 4:**
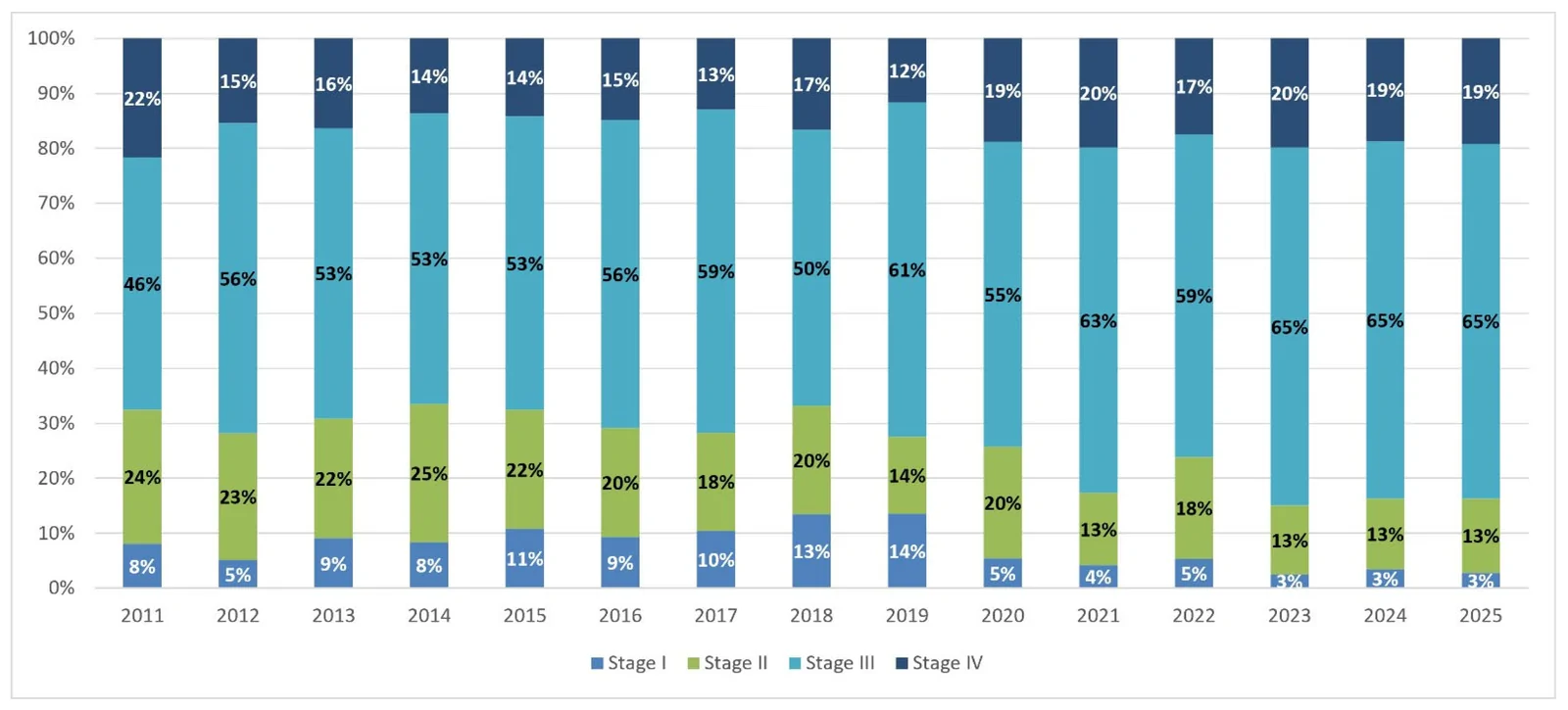
Distribution of colorectal cancer cases by tumor stage at diagnostic in institutional cohort, 2011–2025.

The annual stage distribution showed that advanced-stage disease accounted for most colorectal cancer cases in most years (Figure 4). Although year-to-year variation was observed, the overall pattern indicated a progressive increase in the proportion of stage III–IV cases over time.

Stage III represented the largest proportion of cases in most years, generally accounting for approximately one-half to two-thirds of annual diagnoses. This pattern indicates that a substantial proportion of patients are diagnosed at a locally advanced stage, often involving regional lymph node metastasis.

Stage IV cases also remained consistently present, usually representing around 12–22% of cases per year. In contrast, stage I disease accounted for a small proportion of annual diagnoses and became particularly uncommon in the most recent years.

Stage II represented other common category, with proportions generally ranging between 13% and 25%. In the earlier years (2011–2014), stage II cases accounted for around 22–25%, followed by a gradual decline in subsequent years, stabilizing at approximately 13–18% after 2020.

Overall, 801 patients (22.9%) were diagnosed with stage I–II disease, whereas 2696 patients (77.1%) presented with stage III–IV disease.

The proportion of advanced-stage colorectal cancer increased progressively across the three study periods, from 67.7% in 2011–2015 to 71.1% in 2016–2020 and 82.0% in 2021–2025 (Table 4). This temporal change was statistically significant in both the overall comparison between periods (χ^2^ = 70.190, df = 2, *p* < 0.001) and the linear trend analysis (Linear-by-Linear Association = 65.432, df = 1, *p* < 0.001).

Because stage III and stage IV disease differ in prognosis and treatment intent, stage IV was also analyzed separately. Stage IV disease accounted for 65/434 (15.0%) cases in 2011–2015, 152/1015 (15.0%) in 2016–2020, and 386/2048 (18.8%) in 2021–2025 (Pearson χ^2^ *p* = 0.012; *p* for linear trend = 0.007). The stage III–IV composite should therefore be interpreted as a descriptive measure of disease extent rather than as a proxy for non-curable disease. In sensitivity analyses using calendar year as the ordered variable across all fifteen years, advanced-stage disease increased significantly (*p* < 0.001), as did unspecified grade (*p* < 0.001), confirming that the findings were not dependent on the five-year grouping.

We examined whether advanced stage at diagnosis was associated with sex, early-onset status, urban or rural residence, and tumor location. (Table 5).

Advanced-stage disease was frequent in both sexes accounting for 78.1% of cases among men and 75.5% among women; this difference was not statistically significant (chi-square test, *p* = 0.077).

Area of residence was significantly associated with stage group at diagnosis. Advanced-stage disease was observed in 80.1% of rural residents compared with 75.1% of urban residents, indicating a higher proportion of late-stage presentation among patients from rural areas (chi-square test, *p* = 0.001).

A significant difference was observed according to early-onset status. Advanced-stage disease was recorded in 62 early-onset patients (14.7%) and in 2634 later-onset patients (85.6%) (chi-square test, *p* < 0.001).

Stage distribution was similar across anatomical location groups, with advanced-stage disease accounting for 77.0% of right-sided colon cancers, 77.2% of left-sided/unspecified colon cancers, and 77.1% of rectosigmoid junction/rectal cancers. No statistically significant association was observed between anatomical location and stage group (chi-square test, *p* = 0.996).

All proportions refer to patients treated at this center; their interpretation in relation to the regional population is addressed in Section 4.

### 3.5. Histopathological Grade and Reporting Patterns

**Figure 5:**
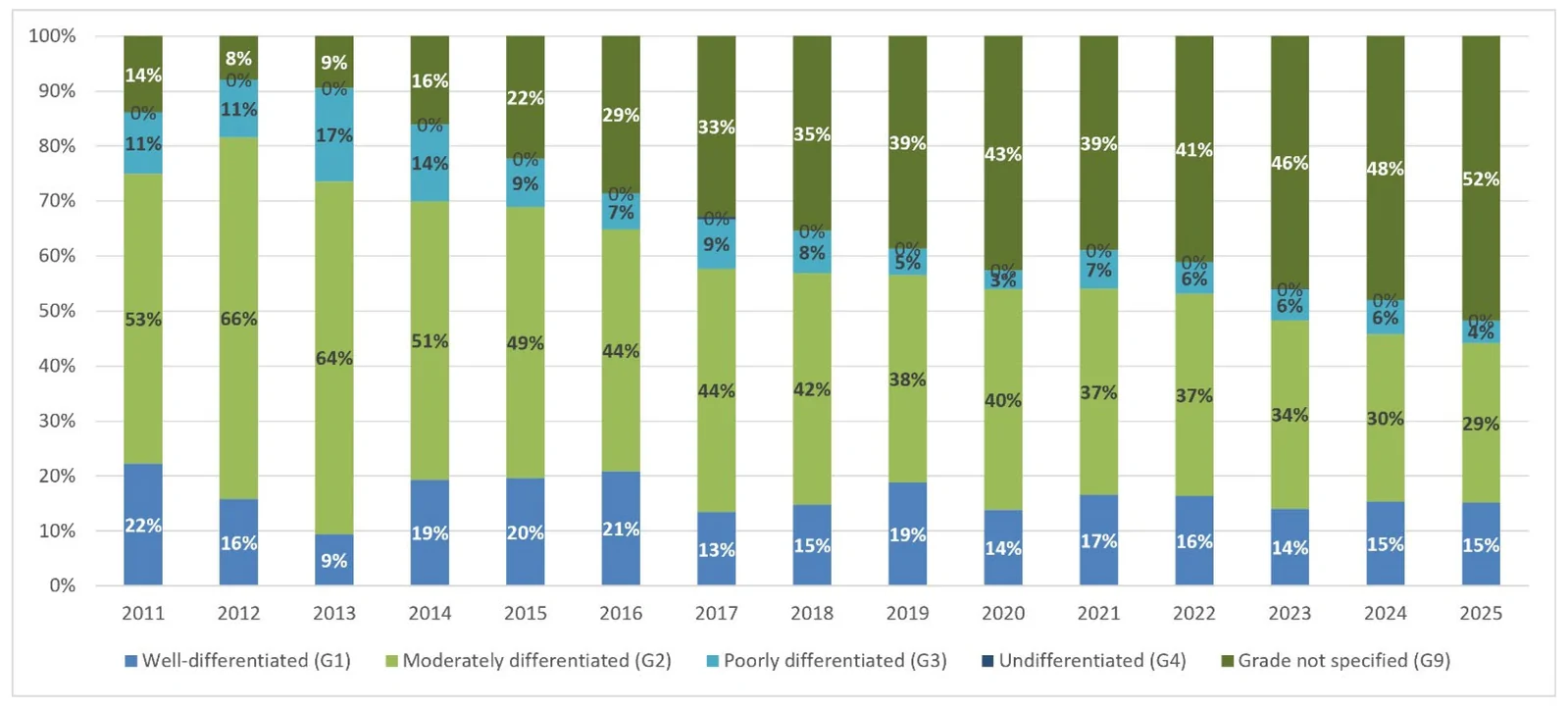
Annual distribution of histopathological grade in institutional cohort, 2011–2025.

The distribution of colorectal cancer cases according to histopathological grade over the period 2011–2025 showed that moderately differentiated tumors were the most frequent documented category, while a substantial proportion of cases had unspecified grade (Figure 5).

Moderately differentiated tumors (Grade 2) represented a large proportion of cases in the earlier years of the study, accounting for approximately 49% to 66% of cases between 2011 and 2015. The highest proportion was observed in 2012 (66%), followed by a gradual decline became evident. From 2016 onward, the proportion of moderately differentiated tumors decreased, reaching approximately 29% by 2025. This pattern indicates a relative decrease in the proportion of documented intermediate-grade tumors over time.

Poorly differentiated and undifferentiated tumors were grouped as high-grade tumors, whereas cases coded as Grade 9 were classified as unspecified grade. In contrast, tumors with unspecified grade increased over time, particularly after 2016, becoming the largest category in the most recent years. This increase should be interpreted primarily as a change in grading documentation rather than as direct evidence of a higher burden of biologically aggressive tumors.

Histopathological grade distribution differed significantly across the three study periods (chi-square test, *p* < 0.001) (Table 6). Low-grade tumors, defined as G1–G2, accounted for 1896 cases (54.2%) overall, while high-grade tumors, defined as G3–G4, represented 237 cases (6.8%). The remaining 1364 cases (39.0%) had unspecified histopathological grade. The proportion of documented low-grade tumors decreased from 71.2% in 2011–2015 to 57.8% in 2016–2020 and 48.8% in 2021–2025. In contrast, tumors with unspecified grade increased from 16.1% in 2011–2015 to 35.8% in 2016–2020 and 45.5% in 2021–2025, suggesting increasing variability or incompleteness in pathological grade reporting over time (Table 6).

### 3.6. Colorectal Cancer Among Digestive Malignancies

Colorectal cancer accounted for the largest share of digestive malignancies recorded at the center throughout the study period, representing between 46% and 82% of cases annually (Figure S1). Gastric and pancreatic cancers followed, each contributing between 11% and 19% in most years, while esophageal, small intestine, and liver and bile duct cancers accounted for smaller and more variable proportions.

## 4. Discussion

### 4.1. Principal Findings

Four findings are directly supported by our institutional dataset: the case volume in our center increased across the three study periods; the cohort showed stable male and urban predominance; the anatomical distribution remained predominantly distal; and the proportions of patients referred with stage III–IV disease and of tumors with unspecified grade increased. Explanations for these observations require confirmation using population-based data.

The findings of this study provide an institutional perspective on colorectal cancer presentation and management in the South-West Oltenia region. Because the data derive from a regional referral oncology center, the results reflect real-world referral patterns, diagnostic pathways, and institutional case volume rather than population-based incidence.

The predominance of advanced-stage disease in this cohort must be interpreted in light of the referral-center design, which is likely to have influenced the case-mix in ways that cannot be separated from any underlying change in the region. Two mechanisms operate in the same direction. First, an oncology center providing systemic treatment necessarily receives those patients for whom such treatment is indicated, and the proportion of referrals meeting that description is expected to have grown as the institution consolidated its regional role and its capacity for multidisciplinary management of complex disease. Second, and less often considered, patients whose early-stage disease is treated definitively by surgical resection may never enter the oncological pathway at all, since adjuvant treatment is not indicated in most stage I and in selected stage II disease; such patients are therefore systematically under-represented in an institutional series of this kind, and increasingly so as surgical management of early disease has become more standardized.

The lower proportion of advanced-stage disease among patients aged < 50 years in this institutional cohort remains unexplained and differs from findings reported in some early-onset colorectal cancer cohorts. Because onward referral and other age-related selection mechanisms were not recorded, this observation should not be generalized to the regional population and requires confirmation using population-based data. One plausible mechanism, advanced as a hypothesis, is the differential patient mobility: younger patients diagnosed with advanced disease may be more likely than older patients to seek care at the larger national oncology centers, whether for a second opinion, for access to clinical trials, or for complex multimodal management such as metastasectomy or specialized surgical intervention. Older patients with advanced disease are, by contrast, more likely to receive systemic and supportive treatment locally. Selective outward referral of this kind would deplete precisely the early-onset, advanced-stage group within an institutional cohort, while leaving the underlying regional distribution unchanged.

Both mechanisms enrich the cohort for advanced disease without any corresponding change in the stage at which colorectal cancer is diagnosed in the population, and both would be expected to intensify over the study period, in parallel with the growth in referral volume documented here. The proportions we report should therefore be read as describing the population referred to and treated at this center, and not as evidence that stage at diagnosis deteriorated across South-West Oltenia. Distinguishing an institutional case-mix effect from a genuine population-level stage migration would require registry data with a defined denominator and complete case ascertainment for the region, neither of which was available for the period studied.

### 4.2. Interpretation in the Context of Existing Evidence

The male predominance in our cohort (60.5%) is consistent with the higher incidence of colorectal cancer among men reported internationally [22,23]. Because the present dataset did not include relevant behavioral or biological exposures for the entire study period, we do not pursue mechanistic explanations.

The predominantly distal distribution of tumors in our cohort, with rectal and sigmoid cancers consistently outnumbering proximal lesions, is consistent with a diagnosis pathway driven by clinical symptoms rather than endoscopic screening, since organized screening programs using colonoscopy have been shown to increase the detection of asymptomatic proximal lesions [24,25].

The advanced-stage presentation documented in our study is a well-recognized contributor to poor outcomes in Romanian colorectal cancer patients. A historical cohort of 302 colorectal cancer patients from a Romanian tertiary center reported an overall 5-year survival of only 38%, substantially below the 55–60% observed in Western European registries, with more than 60% of patients diagnosed at stage III or IV [26]. This pattern of late diagnosis has persisted over time; colorectal cancer mortality in Romania remains 35% higher than the European average in men and 13% higher in women [26,27,28]. The link between diagnostic delay and survival deficit is compounded by broader socioeconomic inequalities. According to International Agency for Research on Cancer (IARC) [29], total cancer mortality rates in Romania vary substantially by educational level: men with primary education have more than double the mortality of those with tertiary education (841 vs. 380 per 100,000), and this social gradient is particularly pronounced for colorectal and stomach cancers. Screening participation in Romania remains among the lowest in the European Union, with population-based colorectal cancer screening still in its early stages [29].

The urban predominance observed in our data (60.3% of cases) exceeds the urban share of the source population by more than ten percentage points: the South-West Oltenia region is predominantly rural, with 49.8% of inhabitants living in urban areas and only 48.6% among those aged 50 years and over. Urban residents are therefore over-represented in our cohort relative to the population from which it is drawn. This is more plausibly explained by differential access to diagnostic services and referral than by a true difference in disease incidence, particularly since rural populations in the ROCCAS pilots demonstrated equal or higher screening engagement when offered access through primary care [15].

The higher proportion of advanced-stage disease among rural residents suggests potential delays in diagnostic access, specialist referral, and completion of colonoscopy evaluation. However, because the study did not include individual data on time to diagnosis, socioeconomic status, screening participation, or distance to care, these explanations should be viewed with caution.

Taken together, these observations suggest that the patterns documented in this cohort are shaped less by distinctive biological factors than by structural characteristics of the healthcare system. The urban predominance and the late-stage distribution appear to be manifestations of the same underlying barriers—limited access to early diagnostics, a social gradient in the use of preventive services, insufficient screening coverage, and variability in diagnostic standardization—which are also those associated with Romania’s persistently elevated cancer mortality.

### 4.3. Histopathological Grading and Reporting Practice

The findings related to histopathological grading further reinforce the notion of variability in diagnostic practices. The high proportion of tumors lacking clearly defined grading highlights potential inconsistencies in reporting or documentation. While this does not necessarily affect immediate clinical management in all cases, it has important implications for epidemiological research and the comparability of data across institutions and regions.

In our cohort, the relatively high proportion of tumors classified as “grade not determined” is most likely related to the intrinsic limitations of endoscopic biopsy specimens used for the initial histopathological assessment of colorectal cancer. Tumor grading in colorectal adenocarcinoma requires adequate preservation of tissue architecture and sufficient viable tumor material; however, endoscopic biopsies frequently provide only small, superficial, or fragmented samples that may not accurately reflect the heterogeneity of the entire lesion [30,31]. In advanced colorectal tumors, extensive necrosis, inflammation, hemorrhage, and crush artifacts can further compromise tissue quality, making reliable evaluation of glandular differentiation difficult or impossible [32]. Moreover, biopsy samples often contain limited invasive tumor tissue, while superficial portions may be overrepresented, increasing the risk of sampling bias and false-negative or inconclusive pathological interpretation [30]. These limitations are well recognized in the literature as a disadvantage of preoperative endoscopic biopsy in colorectal cancer and may explain the elevated percentage of cases with undetermined tumor grade observed in our study population.

Several institutional factors bear on the interpretation of this trend. The study center does not operate its own pathology laboratory; all histopathological examinations were performed in external laboratories, in the surgical and endoscopy units where the diagnostic material was obtained, and reports reached the center as printed documents presented by the patient. Complete reports were required for registration and were requested again from the referring unit where the document initially presented was incomplete. The cases recorded as ungraded therefore correspond to reports in which the issuing laboratory did not state a differentiation grade, and not to incomplete transfer of the pathology document to the center.

This places the finding within reporting practice rather than information flow. Most of the referring laboratories did not use standardized synoptic reporting templates for colorectal carcinoma at any point during the study period; reports were issued in free-text form according to the conventions of each unit. In a narrative report, a grade that cannot be assessed on the material examined is simply not mentioned, and the recipient cannot distinguish an unassessable grade from an omitted one; a synoptic template, by contrast, requires the field to be completed as not assessable, so that the absence of a determination is itself recorded. The absence of a stated grade in our cohort is therefore most plausibly attributable to the nature of the specimen examined—endoscopic biopsies frequently providing material insufficient for reliable assessment of glandular differentiation—combined with the absence of any requirement to record grade as not assessable when it could not be determined. The progressive increase in ungraded reports thus reflects the reporting conventions of the regional pathology network rather than the completeness of documentation available to the treating center, or a change in the quality of histopathological assessment.

In the present cohort, the main temporal change in histopathological reporting was the increasing proportion of tumors for which no grade was specified, which rose from 16.1% in 2011–2015 to 45.5% in 2021–2025. Because the proportion of ungraded reports nearly tripled, the denominator of graded tumors changed substantially between periods, and apparent changes in the relative frequency of low-grade and high-grade tumors cannot be interpreted as reflecting changes in tumor biology. No conclusion is therefore drawn here regarding temporal trends in tumor differentiation. The increase in unspecified grade may reflect incomplete transfer of pathology information, differences between biopsy and resection specimens, or changes in referral and reporting practices; however, these factors were not recorded consistently in the institutional dataset and their individual contributions could not be quantified. The interpretable finding is therefore the change in reporting completeness itself, which carries implications for risk stratification and for the comparability of case series across centers, rather than any inferred change in the biological aggressiveness of the tumors diagnosed.

### 4.4. Screening Context and the Regional Pilot Program

The persistent predominance of advanced-stage disease observed in our cohort, with stage I diagnoses declining to as low as 3% in the final years of the study period, occurred in a setting where organized colorectal cancer screening was largely absent, and was not modified by its recent introduction. The first organized screening pilot programs (The Colorectal Cancer Screening Program in Romania- ROCCAS) were launched in four regions between 2022 and 2023, using a two-step strategy in which general practitioners assessed individual risk via a questionnaire and offered fecal immunochemical testing (FIT) to average-risk individuals aged 50 to 74 [15]. These pilots achieved a FIT return rate of 90%, and notably, rates were higher in rural (91.8%) and vulnerable populations (91.2%) than in urban areas (88.4%), suggesting that GP-mediated outreach can overcome geographic barriers to screening participation [15]. However, the colonoscopy acceptance rate among FIT-positive individuals was only 51.3%, with regional variation and significant delays in scheduling, which represents a critical bottleneck in the screening cascade [15]. These figures describe the screening pathway rather than the pathway through which our patients were identified. The pilot became operational in 2022, within the final years of the observation window; its target range of 50–74 years excludes early-onset disease; and screen-detected cases are recorded in the national screening registry and follow a separate diagnostic route. The patients analyzed here presented symptomatically and were referred for oncological treatment. The program described above therefore provides the context in which the stage distribution we report was generated—one of near-absent early detection—but cannot account for the temporal pattern within our cohort.

The present design cannot estimate the effect of organized screening on stage distribution. The ROCCAS pilot overlapped only with the final years of follow-up, screening status was unavailable in the institutional record, and referral patterns changed over time. Although FIT return was high, colonoscopy completion after a positive FIT remained limited [15]. Longer follow-up with linkage between screening and cancer-registry data will be required to assess a stage shift.

Until 2024, Romania and Bulgaria were the only two EU Member States without a national colorectal cancer screening program; Romania relied instead on pilot projects (ROCCAS) [13]. This longstanding screening gap is the most likely structural explanation for the persistently high proportion of late-stage diagnoses and the declining proportion of stage I cases observed in our cohort. The absence of a national program is reflected in the Organisation for Economic Co-operation and Development (OECD) 2025 country profile, which reports that avoidable CRC mortality in Romania is 57% higher in men and 32% higher in women than the EU average [12].

The screening gap in Romania is best understood in the regional context, where CRC incidence and mortality are among the highest in the EU, yet screening implementation has been heterogeneous. Hungary, with the highest CRC mortality in Europe, launched a nationwide FIT-based program only in December 2018, after pilots achieving 46–51% participation and 57% colonoscopy acceptance among FIT-positive individuals [33,34]; however, public awareness remained low and initial participation stagnated [35]. Serbia introduced organized FIT-based screening earlier, in December 2012, for adults aged 50–74, with a 62.5% response rate in the first round (2013–2014) and 43.3% colonoscopy acceptance, yet approximately 35% of patients still present with metastatic disease [36]. Bulgaria similarly lacked a national CRC screening program for many years, with utilization rates of approximately 4% [37]; in 2025 it launched a national program targeting ages 45–75 with FIT and home-based sampling, informed by a 2024 pilot that screened nearly 100,000 individuals in three months [38]. These comparisons underscore that while neighboring countries have made uneven progress, the shared barriers—low awareness, limited colonoscopy capacity, and suboptimal follow-up—persist across the region, and none has yet achieved participation levels sufficient to shift population-level stage distribution [14].

In a landmark European study of 3.1 million colorectal cancer patients across 21 countries, the divergent trends in incidence, mortality, and stage distribution were largely explained by the level of screening implementation [5]. Countries with long-standing screening—Austria (guaiac fecal occult blood test—gFOBT since 1980, colonoscopy since 2005), the Czech Republic (gFOBT since 2000, organized FIT since 2014), Germany (opportunistic gFOBT since 1977, colonoscopy since 2002, organized since 2019), England (organized gFOBT since 2006, sigmoidoscopy since 2013), and Finland (organized gFOBT since 2004)—showed substantial incidence declines (average annual change −2.5% to −1.3% in men) and clear shifts toward earlier stage at diagnosis. Countries that implemented screening later with rapid, high-coverage rollout—Belgium (organized FIT from 2009–2016 by region), Denmark (2014), Ireland (2012), Lithuania (2009), the Netherlands (2014), and Slovenia (2009)—experienced an initial incidence spike followed by subsequent declines. Conversely, countries with no large-scale screening programs during the study period—Bulgaria, Estonia (pilot FIT from 2016), Norway (trial from 2012), Sweden (regional only), and Ukraine—showed steadily rising incidence (up to +1.9% per year in men), no improvement in stage distribution, and only modest mortality reductions [5]. Romania was not included in this study because it lacked a nationwide cancer registry and organized screening infrastructure during the study period, an absence that underscores the systemic gap reflected in the late-stage predominance observed in our cohort.

The COVID-19 pandemic severely disrupted colorectal cancer screening and diagnostic services worldwide, leading to a documented decrease in new diagnoses and a shift toward more advanced stage at presentation in many countries [39,40]. The largest multicenter study on this topic, including 17,938 patients across 81 Italian centers, found that the pandemic period was independently associated with an increased risk of advanced-stage CRC at diagnosis (OR 1.07, 95% CI 1.01–1.13), aggressive tumor biology (OR 1.32), and stenotic lesions requiring emergency surgery (OR 1.15) [40]. Similarly, the Italian COVID-DELAY study reported a 29% reduction in new CRC diagnoses and a significant drop in early-stage (I–III) presentations from 78% to 63%, with a corresponding rise in stage IV disease from 22% to 37% [39]. In Japan, a hospital-based registry study documented a 34% decrease in stage I CRC diagnoses and a 68% increase in stage III disease after the pandemic onset [41]. In the UK, Shinkwin et al. observed a significant increase in emergency presentations and T4 cancers [42], while Alrahawy et al. reported higher rates of metastatic disease and large bowel obstruction during lockdown periods [43]. The Dutch national screening suspension led to a decrease in CRC incidence largely driven by missed stage I cancers, with 48% of the deficit attributable to early-stage disease [44].

Importantly, in our cohort, the pandemic years (2020–2021) did not introduce a distinct additional worsening beyond the pre-existing trend of late-stage predominance: stage I diagnoses were already low before the pandemic (5–14% in 2011–2019) and remained low through the pandemic period, while stage III disease continued its gradual increase. This absence of a detectable pandemic-specific shift likely reflects the fact that organized screening coverage in Romania was already minimal or absent during the study period, so the pandemic did not create a new bottleneck but rather compounded an existing one. Nevertheless, the international experience underscores that disruptions to screening and diagnostic pathways can rapidly translate into more advanced disease at presentation and poorer outcomes, reinforcing the urgency of establishing resilient, population-based screening programs.

Expanding the ROCCAS model to the national level and addressing the colonoscopy acceptance gap will be essential to reverse the trend of late-stage diagnosis documented in this study. More recently, a 2025 Delphi consensus by 66 international experts (SCREECO) proposed a tailored CRC screening framework for Romania, recommending risk stratification from age 35, screening initiation at age 45, and region-specific strategies (colonoscopy-led for urban areas, FIT-based for rural populations), along with a centralized invitation and recall system [13].

### 4.5. Novelty and Strengths

The principal contribution of this study is a 15-year institutional description of colorectal cancer case-mix in South-West Oltenia, including stage at oncological referral, anatomical subsite, and the completeness of histopathological grade reporting. These data complement, but do not replace, national statistics and population-based registry data.

### 4.6. Limitations

Several limitations of this study should be acknowledged. First, its single-center, retrospective design limits the generalizability of the findings to the entire Romanian population. Although the center serves as a referral unit for South-West Oltenia, referral patterns may introduce selection bias and may themselves have changed over the study period, so that the proportional distributions reported may not reflect the true epidemiological landscape of CRC at regional or national level. Second, the absence of a population denominator precludes the calculation of age-standardized incidence rates and prevents direct comparison with population-based registries, restricting the analysis to proportional distributions rather than absolute risk estimates. Third, data quality and completeness varied across the study period; the proportion of cases classified as “colon, unspecified” and the increasing percentage of tumors with undetermined histopathological grade reflect gaps in diagnostic documentation and reporting standardization—limitations that are well recognized in retrospective cancer registry research.

Fourth, a number of variables essential to the interpretation of our findings were unavailable in the institutional record. No data on treatment modality, treatment response, recurrence or survival were analyzed, and the cohort was not linked to mortality records, which limits the clinical interpretation of the stage distributions. Individual-level information on screening participation, socioeconomic status, healthcare access, symptom duration, the interval from first presentation to diagnosis, distance to the nearest endoscopy unit, and referral route was likewise unavailable; the urban-rural classification was based solely on place of residence at diagnosis and does not capture these dimensions or migration patterns, which may confound the observed disparities. The mechanisms proposed to explain differences between urban and rural patients therefore remain inferential.

Fifth, the study period (2011–2025) spans the COVID-19 pandemic years, during which diagnostic and healthcare disruptions occurred worldwide [39,40]; however, as discussed, the pre-existing trend of late-stage diagnosis in this cohort predated the pandemic, suggesting that the structural screening deficit—rather than a transient pandemic effect—is the dominant driver of the observed patterns. Finally, all analyses were descriptive and unadjusted, relying on univariate trend tests without multivariable adjustment. Age was analyzed as a dichotomous variable at the conventional 50-year threshold for early-onset disease rather than as a continuous variable; while this preserves comparability with the published early-onset literature and with screening eligibility criteria, dichotomization reduces statistical efficiency and does not permit assessment of whether the relationship between age and presentation is graded or threshold-based across the full age range. The associations reported therefore describe differences between groups rather than independent predictors, no causal inference should be drawn from them, and these findings should not be generalized to the regional population.

Despite these limitations, the study provides a valuable longitudinal perspective on colorectal cancer patterns in a region with scarce published data and highlights targets for intervention.

### 4.7. Implications of This Study

The high proportion of stage III–IV disease in this referral cohort supports efforts to shorten diagnostic and referral pathways, particularly for rural patients, while recognizing that the present data cannot establish causality. Standardized pathology reporting would improve the interpretability of tumor-grade data. Linkage of institutional, screening, and population-based registry records is needed to estimate incidence, compare screen-detected with symptomatic disease, and evaluate stage and survival outcomes.

## 5. Conclusions

The present study provides a 15-year real-world perspective on colorectal cancer in South-West Oltenia, Romania, showing a consistent institutional clinicopathological profile characterized by increasing institutional case volume, male and urban predominance, distal tumor localization, frequent advanced-stage diagnosis at oncological referral, and variability in pathological grade reporting.

Colorectal cancer in this regional referral cohort was more frequently observed among male patients and urban residents and was most located in the rectosigmoid junction/rectum and left-sided colon. Although anatomical distribution, sex, and area of residence remained relatively stable over time, the proportion of patients referred with advanced-stage disease increased significantly across the three study periods. In parallel, the growing proportion of tumors with unspecified histopathological grade suggests variability or incompleteness in pathological reporting rather than clear evidence of a biological shift toward higher-grade disease.

These findings support the need for coordinated regional strategies aimed at improving early detection, strengthening referral pathways, broader access to colonoscopy and imaging, and standardizing pathological reporting practices. Although this study does not provide population-based incidence estimates, and its findings describe the population referred to and treated at this center rather than the regional population, it offers relevant real-world evidence for colorectal cancer care planning and screening-oriented interventions in South-West Oltenia.

## Figures and Tables

**Table 1:** Temporal distribution of newly diagnosed colorectal cancer cases across the three five-year study periods.

		n (%) of Total CRC Cohort
Study Period	2011–2015	434 (12.4%)
	2016–2020	1015 (29%)
	2021–2025	2048 (58.6%)
	Total	3497

**Table 2:** Baseline demographic and clinicopathological characteristics of the colorectal cancer cohort.

		n (%)
Gender	male	2115 (60.5%)
	female	1382 (39.5%)
Age at diagnosis	mean ± SD	64.30 ± 11.29
	median	66
Age at CRC Onset	early onset < 50 years	421 (12%)
	late onset ≥ 50 years	3076 (88%)
Area of residence	urban	2108 (60.3%)
	rural	1389 (39.7%)
Tumor localizations	colon, unspecified	383 (11%)
	ascending colon	497 (14.2%)
	descending colon	269 (7.7%)
	sigmoid colon	572 (16.4%)
	transverse colon	157 (4.5%)
	rectosigmoid junction	354 (10.1%)
	rectum	1265 (36.2%)
Stage at diagnosis	I	222 (6.3%)
	II	579 (16.6%)
	III	2093 (59.9%)
	IV	603 (17.2%)
Histopathological grade	well differentiated	560 (16%)
	moderately differentiated	1336 (38.2%)
	poorly differentiated	235 (6.7%)
	undifferentiated	2 (0.1%)
	not specified	1364 (39%)

**Table 3:** Anatomical distribution of colorectal cancer cases across the three study periods.

Anatomical Localization	2011–2015 n (%)	2016–2020 n (%)	2021–2025 n (%)	Total n (%)	*p*-Value *
right-sided colon cancer	86 (19.8%)	181 (17.8%)	389 (19.0%)	656 (18.8%)	0.666
left-sided colon cancer	140 (32.3%)	357 (35.2%)	725 (35.4%)	1222 (34.9%)	
rectosigmoid junction/ rectal cancer	208 (47.9%)	477 (47.0%)	934 (45.6%)	1619 (46.3%)	

* chi-square test.

**Table 4:** Early- and advanced-stage colorectal cancer across the three study periods.

Stage at Diagnostic	2011–2015 n (%)	2016–2020 n (%)	2021–2025 n (%)	Total n (%)	*p*-Value *
Early-stage disease (I–II)	140 (32.3%)	293 (28.9%)	368 (18.0%)	801 (22.9%)	<0.001
Stage III	229 (52.8%)	570 (56.2%)	1294 (63.2%)	2093 (59.9%)	
Stage IV	65 (15%)	152 (15%)	386(18.8%)	603 (17.2%)	

* Pearson chi-square test and Linear-by-Linear Association test for trend.

**Table 5:** Association between demographic and anatomical characteristics and stage group at diagnosis.

		Early-Stage Disease n (%)	Advanced-Stage Disease n (%)	*p*-Value *
Gender	male	463 (21.9%)	1652 (78.1%)	0.077
	female	338 (24.5%)	1044 (75.5%)	
Age at CRC Onset	early onset < 50 years	359 (85.3%)	62 (14.7%)	<0.001
	late onset ≥ 50 years	442 (14.4%)	2634 (85.6%)	
Area of residence	urban	525 (24.9%)	1583 (75.1%)	<0.001
	rural	276 (19.9%)	1113 (80.1%)	
Tumor localizations	right sided	151 (23.0%)	505 (77.0%)	0.996
	left sided	279 (22.8%)	943 (77.2%)	
	rectosigmoid junction/rectum	371 (22.9%)	1248 (77.1%)	

* Pearson chi-square test.

**Table 6:** Histopathological grade distribution across the three study periods.

Histopathological Grade	2011–2015 n (%)	2016–2020 n (%)	2021–2025 n (%)	Total n (%)	*p*-Value *
Low-grade (G1–G2)	309 (71.2%)	587 (57.8%)	1000 (48.8%)	1896 (54.2%)	<0.001
High-grade (G3–G4)	55 (12.7%)	65 (6.4%)	117 (5.7%)	237 (6.8%)	
Unspecified grade	70 (16.1%)	363 (35.8%)	931 (45.5%)	1364 (39.0%)	

* Pearson chi-square test.

## Data Availability

The datasets generated and analyzed during the current study are not publicly available due to institutional confidentiality regulations and the sensitive nature of patient-level medical data. Anonymized aggregated data may be made available from the corresponding author upon reasonable request, subject to institutional and ethical approval.

## Supplementary Materials

The following supporting information can be downloaded at: https://www.mdpi.com/article/10.3390/cancers18162615/s1. Figure S1: Annual distribution digestive malignancies recorded at the oncology centre, 2011–2025.

## Abbreviations

The following abbreviations are used in this manuscript:

AJCC	American Joint Committee on Cancer
CRC	Colorectal cancer
EU	European Union
FIT	Fecal Immunochemical Test
G1–G4	Histopathological grade 1 to 4
Gfobt	Guaiac fecal occult blood test
GLOBOCAN	Global Cancer Observatory
IARC	International Agency for Research on Cancer
ICD-10	International Statistical Classification of Diseases and Related Health Problems, 10th revision
ICD-O-3	International Classification of Diseases for Oncology, 3rd edition
NUTS-2	Nomenclature of Territorial Units for Statistics, level 2
OECD	Organisation for Economic Co-operation and Development
ROCCAS	Romanian Colorectal Cancer Screening Program
SD	Standard deviation
SPSS	Statistical Package for the Social Sciences
TNM	Tumor, Node, Metastasis
