# Supplementary material for: Colorectal Cancer Burden and Trends in South-West Oltenia, Romania: A 15-Year Real-World Study from a Regional Referral Cancer Center
Source: Cancers (Basel). 2026 Aug 13;18(16):2615. doi: 10.3390/cancers18162615 (PMC13510670; doi:10.3390/cancers18162615)
Supplement: Supplementary file 1 [file cancers-18-02615-s001.zip › cancers-4430200-supplementary.pdf]

Supplementary Materials

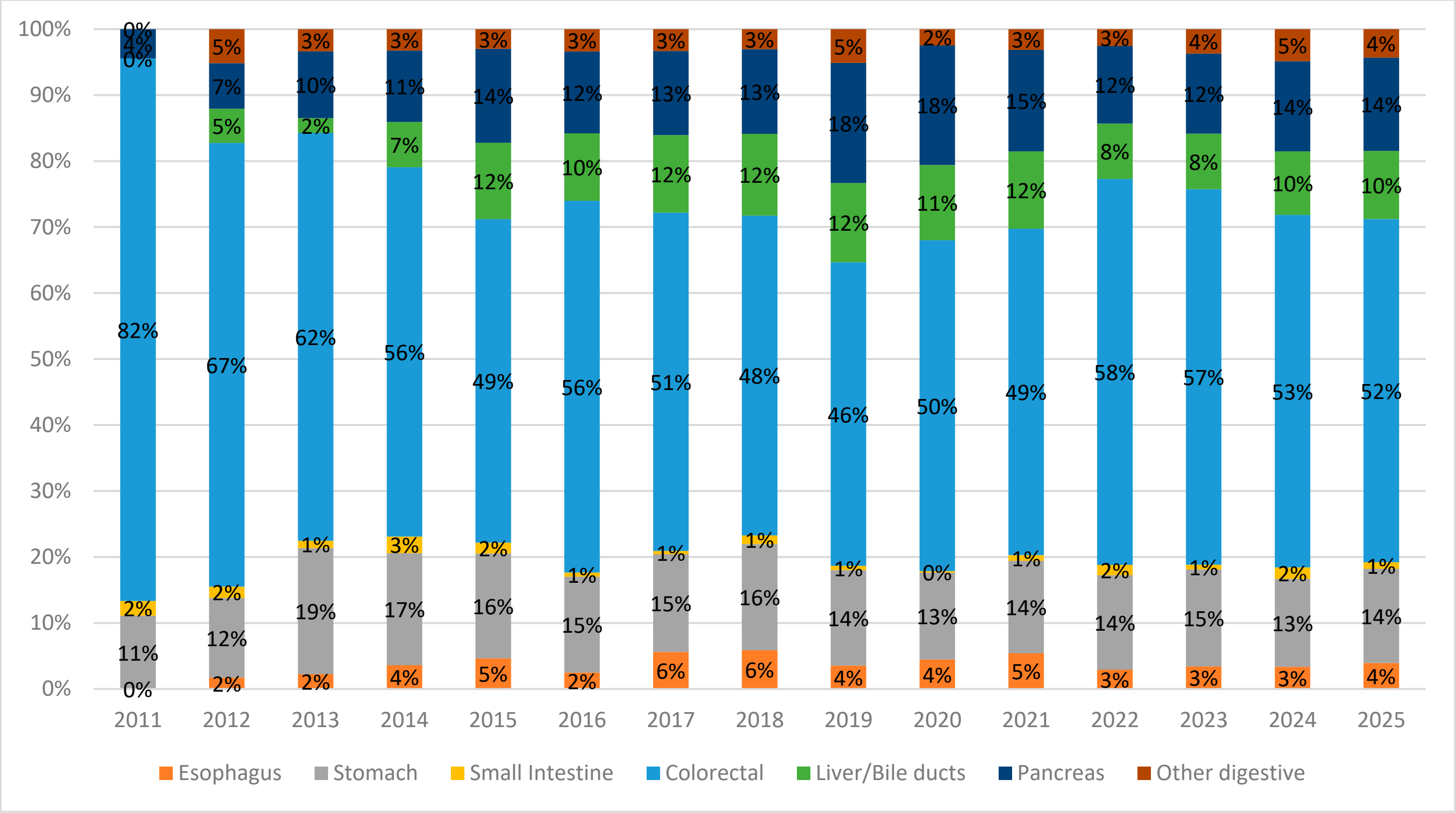

Figure S1. Annual distribution digestive malignancies recorded at the oncology centre, 2011–2025.
